# Effect of Hydrothermal Aging Treatment on Decomposition of NO by Cu-ZSM-5 and Modified Mechanism of Doping Ce against This Influence

**DOI:** 10.3390/ma13040888

**Published:** 2020-02-17

**Authors:** Xiao Yang, Xiaofei Wang, Xiaolei Qiao, Yan Jin, Baoguo Fan

**Affiliations:** College of Electrical and Power Engineering, Taiyuan University of Technology, Yingze West Street 79, Taiyuan 030024, China; yangxiao0165@link.tyut.edu.cn (X.Y.); wangxiaofei0167@link.tyut.edu.cn (X.W.); qiaoxiaolei@tyut.edu.cn (X.Q.); jinyan@tyut.edu.cn (Y.J.)

**Keywords:** Cu-ZSM-5, NO decomposition, hydrothermal aging, {Cu^2+^-O^2−^-Cu^2+^}^2+^, Ce modification

## Abstract

Cu-ZSM-5 and Ce-doped Cu-Ce-ZSM-5 samples were prepared by liquid-phase ion exchange method. The two catalysts were subjected to hydrothermal aging treatment in the simulated flue gas of a coal-fired power station at an ageing temperature of 650–850 °C. The denitration experiment found that the activity of the aged Cu-ZSM-5 was 19.6% to 41% lower than that of the fresh Cu-ZSM-5 at the optimal decomposition temperature of NO at 550 °C, while the aged Cu-Ce-ZSM-5 had only a 14.8% to 31.5% reduction in activity than the fresh Cu-Ce-ZSM-5. The samples were characterized by XRD, BET, H_2_-TPR, XPS, NO-TPD, etc. The results showed that hydrothermal aging treatment leads to the dealumination of the ZSM-5 framework and reduces the specific surface area and pore volume of the micropore in the sample. It also exacerbates the isolated Cu^2+^, and the active center {Cu^2+^-O^2−^-Cu^2+^}^2+^ dimers migrate towards the sample surface and form inactive CuO. Doping with Ce can promote the dispersion of Cu(OH)^+^, which was the precursor of {Cu^2+^-O^2−^-Cu^2+^}^2+^. Ce^3+^ can preferentially occupy the less active bridged hydroxyl exchange sites, so that copper ions occupy the more active aluminum hydroxyl sites, thereby inhibiting the migration of active centers.

## 1. Introduction

The NO from coal combustion is one of the main pollutants in the operation of coal-fired power stations. Direct emissions of NO to atmosphere lead to photochemical smog, acid rain and ozone layer destruction. In addition to affecting the natural environment, NO also causes respiratory, cardiovascular, and cerebrovascular diseases [1,2]. Although most coal-fired power station boilers are equipped with a Selective Catalytic Reduction (SCR) denitration system [3], ammonia escapes, the narrow temperature window, the highly toxic of V_2_O_5_ catalyst, and the blocked downstream air preheater caused by side reactions seriously affects the stability and reliability of SCR [4,5].

The most ideal form of denitration is to directly decompose NO into N_2_ and O_2_, which does not need any reducing agent and will not produce secondary pollution [6]. The NO decomposition reaction is thermodynamically favorable, but the kinetic activation energy is as high as 364 kJ/mol. Finding a suitable catalyst becomes the key to the maturity of this method [7,8]. Catalysts for catalytic decomposition of NO mainly include noble metals, metal oxides, perovskite, and ion-exchanged ZSM-5 zeolite [9,10]. ZSM-5 zeolite has good pore structure and specific surface area which can provide more active sites. Since Iwamoto found that Cu-based zeolite has the ability to directly decompose NO [11], catalytic decomposition of NO used Cu-ZSM-5 has become a research hotspot. At present, Cu-ZSM-5 is the most effective catalyst besides noble metals, but there is still a gap between its catalytic activity and industrial application requirements.

Many scholars have worked to improve the catalytic activity of Cu-ZSM-5 and achieved certain results [12,13], but most of their research has been conducted in relatively mild conditions in the laboratory. In fact, the flue gas generated during the operation of the coal-fired power station boiler contains a certain amount of water vapor. Unlike the optimum temperature between 300–400 °C required for catalytic reduction of NO in SCR system, the activity temperature of the Cu-ZSM-5 used for direct catalytic decomposition of NO should be in 500–600 °C. In order to simulate the long-term hydrothermal aging process of the catalyst, it is suitable for the laboratory to accelerate the aging by increasing the temperature to 650–850 °C based on the fact that no other changes related to the hydrothermal aging treatment occur in this temperature range. At present, there is little research on the real performance of Cu-ZSM-5 under the harsh conditions of simulating coal-fired power station flue gas containing high-temperature water vapor for a long time. However, the phenomenon that the hydrothermal aging treatment causes the deactivation of the zeolite catalyst has been found in the denitration field of automobile exhaust gas. Kwak found that the decrease of SCR activity of Cu-ZSM-5 caused by hydrothermal aging was due to the great change of copper species [14]. Ma found that the NH_3_-SCR activity of Cu-SSZ-13 and Cu-SAPO-34 treated by hydrothermal aging decreased significantly [15]. The expensive catalyst brings great cost pressure to the denitration system of the coal-fired power station. It is important to study the deactivation mechanism of Cu-ZSM-5 under hydrothermal aging treatment conditions and improve its anti-hydrothermal aging performance.

Many studies found that the mechanism of direct catalytic decomposition of NO used Cu-ZSM-5 is relatively consistent. It is believed that the active center is {Cu^2+^-O^2−^-Cu^2+^}^2+^ dimer [16], which decompose NO through a redox reaction [17,18]. Besides, the preparation conditions of the catalyst, such as the type of copper source and the exchange temperature affect the content of the active center, and thus the Cu-ZSM-5 exhibits different catalytic activities [19]. The hydrothermal aging treatment also has effects on the content and stability of the active center of Cu-ZSM-5, but there are few relevant reports. Copper entering the ZSM-5 zeolite through ion exchange will exist in various forms (isolated state, dimer, oxidation, etc.) [20] and will be transformed under certain conditions. When the active copper species is converted to inactive copper species, the activity of the catalyst is naturally decreased. The main idea to improve the hydrothermal stability of the catalyst is to prevent migration, aggregation, and transformation of the active copper species. What is interesting is that the addition of rare earth elements can improve the ability of Cu-ZSM-5 and Cu-SAPO-34 to remove NO without water vapor [21,22].

In this study, the Cu-ZSM-5 and Cu-Ce-ZSM-5 catalysts were prepared by ion-exchange of the ZSM-5 support. The samples were aged in the simulated flue gas at 650, 750, and 850 °C for 10 h respectively. The decomposition efficiency of NO between the fresh and the aged samples was compared, and the samples were characterized by various methods. The deactivation mechanism of the hydrothermal aged catalyst and the modification mechanism by adding Ce to improve the anti-hydrothermal aging property were obtained. The results of this study provide some useful references for the practical application of the method which used Cu-ZSM-5 to decompose NO directly in the denitration field in coal-fired power station boiler.

## 2. Materials and Methods

### 2.1. Preparation of Catalyst

Ammonia exchange is carried out before Cu exchange. The specific preparation steps are as follows: 10 g of H-ZSM-5 is immersed in 0.1 L of a 1 mol/L ammonium nitrate solution. The mixture was stirred and exchanged by a magnetic stirrer at 80 °C for 2 h. After the completion of the stirring, the mixture was suction filtered and washed three times with deionized water, and the solid was dried in a drying oven at 90 °C overnight to obtain NH_4_-ZSM-5. Cu-ZSM-5 was prepared from NH_4_-ZSM-5, and 1 L of a 0.01 mol/L copper nitrate solution was added. Then, the mixture was stirred and exchanged by a magnetic stirrer at 40 °C for 24 h. After the completion of the stirring, the copper ions remaining on the surface of the ZSM-5 zeolite were washed 6 times to prevent the form of CuO during the calcination process. After the washing was completed, the sample was placed in a drying oven to be dried overnight and then calcined in an inert atmosphere at 550 °C for 4 h to obtain a Cu-ZSM-5. The Cu-Ce-ZSM-5 catalyst, which is doped with Ce, is prepared by a common ion exchange method. NH_4_-ZSM-5 exchanged with 0.01 mol/L of copper nitrate and 0.002 mol/L of cerium nitrate in the same procedure as above. Finally, all samples were pelletized, crushed, and sieved to 40–60 mesh for later use.

The flue gas used to simulate the hydrothermal aging process is composed of 5% oxygen, 15% carbon dioxide, 10% water vapor, and 70% nitrogen. The fresh catalysts were hydrothermally aged at 650, 750, and 850 °C for 10 h, respectively, and the samples were named Cu-ZSM-X and Cu-Ce-ZSM-X where X represents the hydrothermal aging temperature.

### 2.2. NO Decomposition Evaluation Experiment

**Figure 1:**
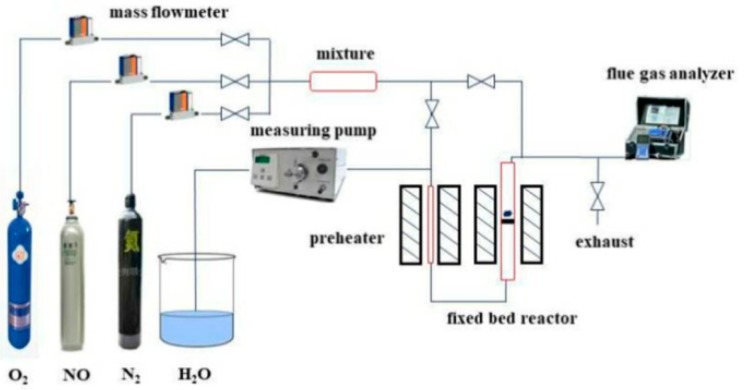
Fixed-bed reaction system for hydrothermal aging treatment and evaluation of NO decomposition activity of catalyst.

The catalytic activity of the sample to decompose NO at different reaction temperatures was tested in a fixed-bed miniature reactor. The experimental system is shown in Figure 1. The inside diameter of the reactor was 10 mm, and the catalyst sample was 0.5 g. The temperature is raised to the reaction temperature at a heating rate of 10 °C/min, the sample is pre-treated in an atmosphere with an N_2_ speed of 20 mL/min for 2 h, and then the gas path is switched to the reaction atmosphere. The catalyst was exposed to a gas flow of 20 mL/min with the following composition: 0.5% NO, 5% O_2_, 10% H_2_O, and N_2_ as the balance gas. When testing the efficiency of the catalyst in the absence of water vapor, the atmosphere becomes: 0.5% NO, 5% O_2_, and N_2_ as the balance gas. The GHSV was about 1200 h^−1^. The concentration of NO was analyzed continuously using a flue gas analyzer (German: ecom-J2KN). 

The catalytic activity of the catalyst is evaluated by the NO conversion rate, and the calculation method is as shown in Formula (1). Where C_0_(NO) is the inlet concentration of NO (mg/m^3^) and C_1_(NO) is the outlet concentration of NO (mg/m^3^).

(1) $$X(\mathrm{NO})=\frac{C_{0}(\mathrm{NO})\text{-}C_{1}(\mathrm{NO})}{C_{0}(\mathrm{NO})}\times 100\%$$

### 2.3. Characterization of the Catalyst

Si, Al, Cu, and Ce elemental content of catalysts were measured by ICP-OES (Inductively Coupled Plasma Optical Emission Spectrometer) on a 730/Agilent 7700 (Agilent Technologies, Santa Clara, CA, USA). The crystal structure was observed by X-ray diffraction on a DX2700B type X-ray powder diffractometer (Dan Dong Hao Yuan Instrument Co., Ltd., Dandong, China). The nitrogen low temperature physical adsorption experiment was carried out on a MicroActive for ASAP 2460 instrument (Micromeritics, Norcross, GA, USA), and the sample was heated at 300 °C for 6 h under vacuum conditions before the test. SEM (Scanning Electron Microscope) was obtained on a JSM-IT200 scanning electron microscope (JEOL Ltd., Akishima, Japan). XPS (X-ray Photoelectron Spectroscopy) was performed on a Thermo Scientific ESCALAB 250Xi spectrometer (Thermo Fisher Scientific, Waltham, MA, USA) using Al Kα as the X-ray source (1486.6 eV) with C1s as the standard and a binding energy error of ±0.05 eV. The detection depth of XPS is approximately 50 to 100 Å, meeting the sensitivity requirements.

The H_2_-TPR (Temperature Programmed Reduction) was performed on an AutoChem 2910 instrument (Micromeritics, Norcross, GA, USA). A 0.2 g sample of the catalyst was placed in a U-shaped quartz tube reactor with an inner diameter of 10 mm and pretreated at 500 °C for 4 h in Ar (15 mL/min). After cooling to room temperature in the same atmosphere, the gas path was switched to 9.96% H_2_/Ar (15 mL/min), and the temperature was increased from room temperature to 800 °C at a heating rate of 10 °C/min.

The NO-TPD (Temperature Programmed Desorption) experiment was carried out on a laboratory-made microreactor. A 0.2 g catalyst was placed in a reactor, treated at 550 °C for 1 h in N_2_ (50 mL/min), and then cooled to room temperature and treated with 0.5% NO/N_2_ (100 mL/min) for 2 h until the flue gas analyzer showed that the NO content no longer changed to ensure that the NO adsorption is complete. Then, the gas path was purged with N_2_ at a flow of 200 mL/min until no NO was detected. Thereafter, the temperature was increased to 550 °C at a temperature increase rate of 10 °C/min to start the desorption experiment. Desorption signal of NO_x_ was detected by a flue gas analyzer.

## 3. Results and Discussion

### 3.1. NO Decomposition Activity of Different Catalysts

**Figure 2:**
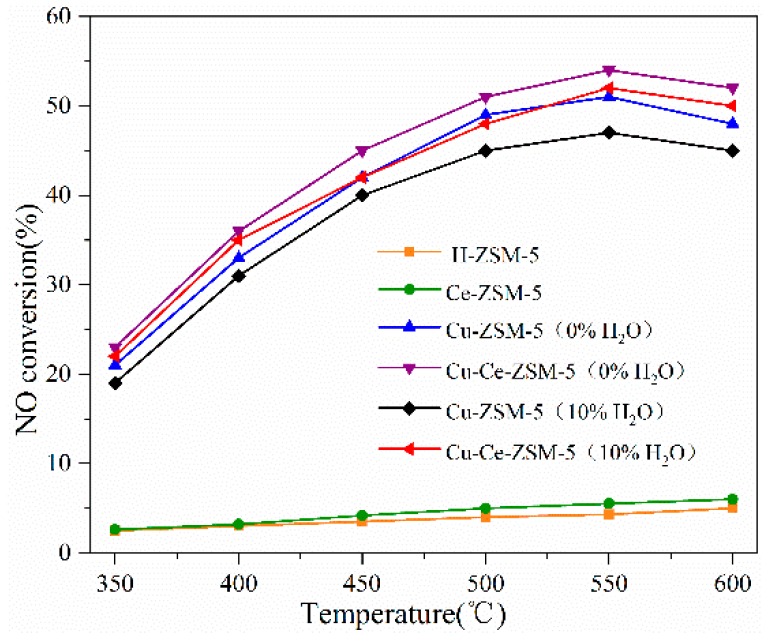
NO decomposition activity of fresh catalyst: Cu-ZSM-5 and Cu-Ce-ZSM-5 were tested in a reaction atmosphere with no water vapor and 10% water vapor, respectively; H-ZSM-5 and Ce-ZSM-5 were tested in a reaction atmosphere that does not contain water vapor.

Figure 2 shows the denitration activity of a fresh catalyst. Compared with Cu-ZSM-5, H-ZSM-5 and Ce-ZSM-5 have substantially no NO decomposition activity, indicating that the copper component in the catalyst is essential. The NO conversion rates of Cu-ZSM-5 and Cu-Ce-ZSM-5 at an optimum reaction temperature of 550 °C in the reaction atmosphere containing 10% water vapor were 47% and 49%. The activity of the catalyst was partially restored when the reaction atmosphere was changed to non-water vapor, and the NO conversion rates were 51% and 54%. This experimental result is similar to that of Iwamoto [23], which indicates that the water vapor in the reaction atmosphere has a toxic effect on the catalyst. Fang studied the interaction between water vapor and catalytic active center {Cu^2+^-O^2−^-Cu^2+^}^2+^ by IR characterization [24]. It was found that water molecules could attack {Cu^2+^-O^2−^-Cu^2+^}^2+^ species at high temperature and make them change to Cu(OH)^+^. The decrease of {Cu^2+^-O^2−^-Cu^2+^}^2+^ species led to a decrease in NO decomposition efficiency. After the water was removed, the {Cu^2+^-O^2−^-Cu^2+^}^2+^ recovered and the denitration activity was also restored.

The Ce modified Cu-Ce-ZSM-5 sample was more active than Cu-ZSM-5 in the reaction atmosphere of no water vapor. When the Ce modified sample was changed from a non-water vapor atmosphere to a water vapor atmosphere, the decrease of NO conversion was lower than that of Cu-ZSM-5, indicating that Ce modification has effect to improve the ability to against water vapor poisoning of Cu-ZSM-5.

**Figure 3:**
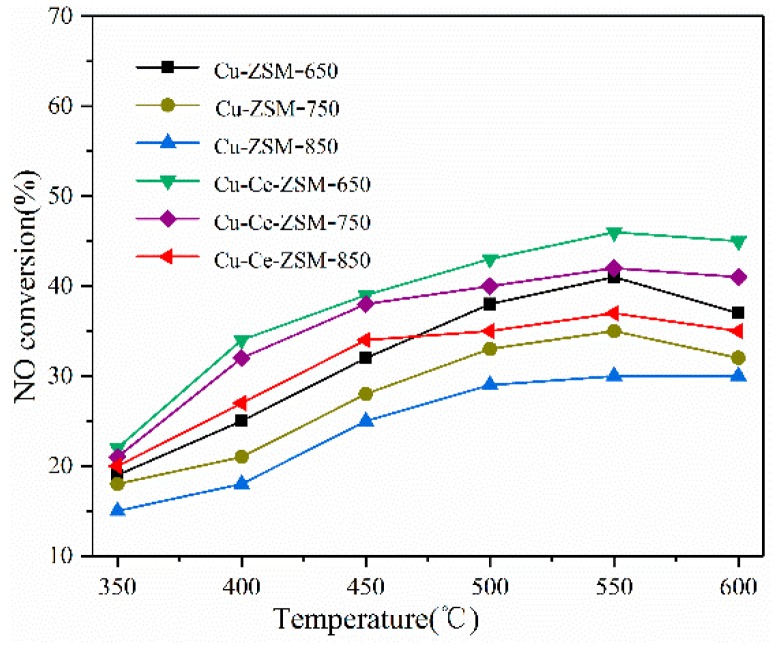
Decomposition activity of Cu-ZSM-X and Cu-Ce-ZSM-X (X: 650, 750, 850 °C) treated with different hydrothermal aging temperatures in a reaction atmosphere containing 10% water vapor.

Figure 3 shows the denitration activity of Cu-ZSM-X and Cu-Ce-ZSM-X catalysts, and the reaction atmosphere always contains 10% water vapor. The NO conversion rates of the Cu-ZSM-X subjected to hydrothermal aging at 650, 750, and 850 °C were maximum at a reaction temperature of 550 °C and were 41%, 35%, and 30%, respectively. Compared with the fresh catalyst in Figure 2, the reaction activity of Cu-ZSM-X was significantly reduced. As the temperature of the hydrothermal aging treatment increased, the amplitudes of the decrease in catalytic activity also increased, which were 19.6%, 31.3%, and 41.2%, respectively. Although the catalytic decomposition activity of NO of the Ce-modified Cu-Ce-ZSM-X is also reduced, it is more active than the Cu-ZSM-X at the same temperature. The NO conversion rates of Cu-Ce-ZSM-X obtained after three different temperatures of hydrothermal aging treatment were 46%, 42%, and 37%, respectively, which were 14.8%, 22.2%, and 31.5% lower than the fresh Cu-Ce-ZSM-5 samples. This indicates that Ce modification could also improve the hydrothermal aging resistance of Cu-ZSM-5.

### 3.2. Characterization Results of the Catalysts

#### 3.2.1. Structural Characteristics

The results of ICP-OES detection were shown in Table 1. The contents of copper in the fresh Cu-ZSM-5 and Cu-Ce-ZSM-5 were basically the same, 1.75% and 1.69%, respectively. The Cu ion exchange degrees calculated by the method that a copper ion exchange two NH_4_^+^ were 51.6% and 49.2%. The ICP-OES results indicated that the two catalysts substantially have the same degree of Cu ion exchange during catalyst preparation. Many studies had shown that the activity of Cu-ZSM-5 to decompose NO increases with the increase of Cu ion exchange degree [11]. The same copper content in this experiment was a prerequisite for conducting an evaluation test for catalytic decomposition NO of catalyst, and the effect of Ce modification could be fully explained.

**Figure 4:**
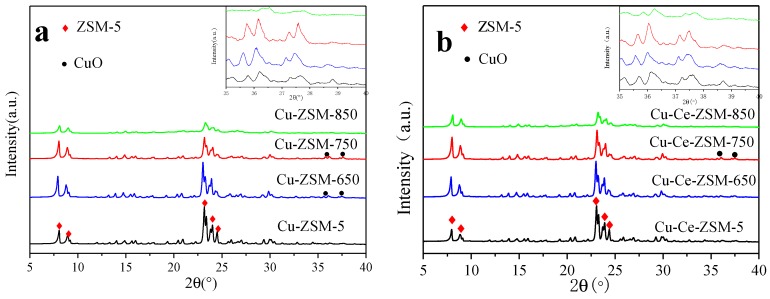
XRD patterns of different catalysts. (**a**) Cu-ZSM-5 and Cu-ZSM-X. (**b**) Cu-Ce-ZSM-5 and Cu-Ce-ZSM-X.

In order to investigate the effect of hydrothermal aging treatment on the framework structure of Cu-ZSM-5 and Cu-Ce-ZSM-5, the samples were characterized by XRD. The results were shown in Figure 4. Figure 4a corresponds to fresh Cu-ZSM-5 and Cu-ZSM-X treated by hydrothermal aging at different temperatures. Figure 4b corresponds to fresh Cu-Ce-ZSM-5 and Cu-Ce-ZSM-X. There were diffraction peaks of the ZSM-5 structure (PDF#44-0003) in all samples [25]. With the increase of hydrothermal aging temperature, the intensity of the diffraction peak corresponding to the ZSM-5 structure in Cu-ZSM-X and Cu-Ce-ZSM-X showed a downward trend. Under the conditions of 650 and 750 °C hydrothermal aging temperature, the diffraction peak intensity of each catalyst decreased slightly, while the 850 °C hydrothermal aging Cu-ZSM-850 and Cu-Ce-ZSM-850 showed significant decline. It was indicated that the zeolite framework begins to be destroyed with the increase of the temperature of the hydrothermal aging treatment and the damage was most at 850 °C. Related to the experimental results that the activity of catalytic decomposing NO decreases with the increase of hydrothermal aging temperature, it indicated that high temperature hydrothermal aging could reduce the catalytic efficiency by destroying the zeolite framework structure. The destruction of the framework structure was mainly caused by dealumination [26]. After dealumination, the silica-alumina ratio of the framework was increased, and the exchangeable active sites were lowered, resulting in a decrease in the activity of the catalyst to decompose NO. The variation tendency of XRD diffraction peak of the Cu-Ce-ZSM-X was almost the same as that of the Cu-ZSM-X. This indicated doping Ce did not improve the strength of the ZSM-5 framework. The increase in catalyst activity was not pertinence of the zeolite structure [27]. It was worth noting that a diffraction peak of CuO (PDF#45-0937) was observed in three samples of Cu-ZSM-650, Cu-ZSM-750, and Cu-Ce-ZSM-750. Moreover, the diffraction peak intensity of the Cu-ZSM-750 was greater than the Cu-ZSM-650, indicating that CuO was gradually formed as the hydrothermal aging temperature increases. No obvious CuO was detected in the modified sample after aged at 650 °C, and a small amount of CuO did not appear until 750 °C, indicating that doping Ce can slow down the formation of CuO. The modified and unmodified samples aged at 850 °C should also exist CuO crystals, but the high temperature of 850 °C caused a serious decrease in the crystallinity of the catalyst, high-temperature may cause CuO to decompose to form Cu_2_O, and the significant CuO diffraction peak was not observed. In addition, diffraction peaks of oxides related to Cu and Ce were not observed in other aged samples, but it did not mean that there is no corresponding oxide. According to the results of ICP-OES, it could be found that the relative contents of the two elements in the samples were relatively low, 1.75%, 1.69%, and 0.027%, respectively. Indicating that the copper and cerium oxides are well dispersed as amorphous metal species or aggregated into mini-crystals that are too small (<3 nm) to be detected by XRD [22].

SEM was used for the purpose of explaining the surface morphology changes of the modified and unmodified samples under different hydrothermal aging temperature. (The SEM images (Figure S1.) and explanations are provided in the Supplementary Materials).

In order to investigate the effect of hydrothermal aging treatment on the pore structure of Cu-ZSM-5 and Cu-Ce-ZSM-5, two fresh samples and some aged samples were characterized by low temperature nitrogen physical adsorption. The adsorption–desorption curves are shown in Figure S2. (The figure and its explanations are provided in the Supplementary Materials.).

The specific surface area, pore volume and pore diameter results of fresh samples and hydrothermally aged samples were shown in Table 2. The total specific surface areas of fresh Cu-ZSM-5 and Cu-Ce-ZSM-5 were 316.6 and 309.5 m^2^/g. The specific surface area of Cu-ZSM-650 and Cu-Ce-ZSM-650 increased slightly compared with fresh samples, while that of the samples treated at 750 and 850 °C decreased. The decrease amplitude of the 750 °C aged sample was smaller than 850 °C. The decrease proportion of Cu-ZSM-850 and Cu-Ce-ZSM-850 reached 43.1% and 42.9%. Although the variation trends of the total specific surface area of the samples after hydrothermal aging were inconsistent—it rose first and then fell—the total specific surface area of the sample treated by 650 °C did not fall but increased. However, the variation trends of the area of micropore in all samples after hydrothermal aging were consistent and showed a downward tendency. Reduction range of the unmodified Cu-ZSM-X was from 58.3% to 65.6%, and that of the modified Cu-Ce-ZSM-X was from 55.4% to 64.7%.

The pore volumes of the micropore in the fresh Cu-ZSM-5 and Cu-Ce-ZSM-5 were 0.121 and 0.120 cm^3^/g. The reduction range of pore volumes in Cu-ZSM-X was from 54.5% to 66.1%, and the modified Cu-Ce-ZSM-X decreased range was from 51.6% to 65.0%. The variation trend of micropore volume is similar to that of micropore specific surface area. The decrease of the micropore area and volume was caused by the destruction of the ZSM-5 zeolite by the high temperature water vapor, which caused the framework aluminum to fall off. The higher the hydrothermal aging temperature, the more serious the framework aluminum detachment. A large amount of non-framework aluminum would accumulate in the pores of the zeolite to cover and block the micropore [26].

The specific surface areas of the fresh Cu-ZSM-5 and Cu-Ce-ZSM-5 mesopore were 75.4 and 71.5 m^2^/g, while Cu-ZSM-650 and Cu-Ce-ZSM-650 was up to 224.0 and 219.2 m^2^/g. The increased area of mesopore was larger than the area of micropore reduction. Therefore, it could be explained that the total specific surface area of the sample treated at 650 °C was larger than that of the fresh sample. The adsorption–desorption curves of all aged samples showed obvious hysteresis loops at p/p_0_ = 0.45, which proved the existence of mesopore. The presence of mesopore was due to the high temperature water vapor treatment that caused a fracture in the framework of ZSM-5. The micropore connected to each other and mesopore were formed. The higher the temperature of hydrothermal aging, the stronger this process was, and new mesopore with larger pore diameter was formed by connected between the mesopore. Figure S3 (see in Supplementary Materials) showed the pore diameter distribution of the fresh and hydrothermal aged samples. In addition to micropore, there were also mesopores with a wide pore size range between 10 and 40 nm. The size of the hysteresis loop that occurs in the formation of mesopore in Figure S2 could also characterize the pore diameter of the mesopore. It was found that the pore diameter of the 850 °C aged sample was larger than 650 and 750 °C aged samples. The formation of new mesopore with larger pore diameter caused decrease in the total number of mesopores, which also corresponds to the results of the sharp reduction in the specific surface area of mesopore at 850 °C in Table 2. It is illustrated that the high-temperature hydrothermal conditions at 850 °C damage the ZSM-5 framework more seriously, which is consistent with the XRD results that the diffraction peak intensity of 850 °C treated sample was significantly reduced. The average pore size of the mesopore of all samples gradually increased, from 2.145 to 2.664 nm of the unmodified sample and from 2.124 to 2.629 nm of the modified sample.

**Figure 5:**
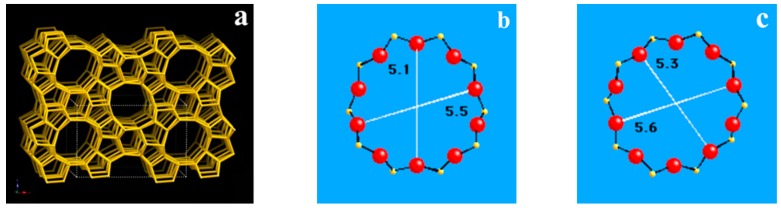
Schematic diagram of the framework structure and pore size of ZSM-5 zeolite. (**a**) Framework structure of ZSM-5. (**b**) 10-ring pore diameter viewed along [100]. (**c**) 10-ring pore diameter viewed along [010].

There was a typical shape-selective characteristic of Cu-ZSM-5 in the catalytic decomposition of NO. The exchange sites and active sites were all in the pores inside the zeolite. The pores of the ZSM-5 were three-dimensional intersecting channels composed of straight and sinusoidal channels with a pore size of 5.1 to 5.6 Å, all of which were micropores, as shown in Figure 5. Although with the increase of hydrothermal aging temperature, the proportion of specific surface area contributed by mesopore decreased gradually, and the proportion of micropore increases gradually; the absolute area and pore volume of micropore are gradually reduced. The variation trends of the specific surface area and pore volume of the micropore in all the samples were also consistent with the evaluation results. It was also proved that the specific surface area of the micropore was the main factor in the catalyst pore structure aspect to influence the catalytic activity.

BET and XRD results showed that the pore structure of the catalyst could not be effectively improved after the Ce modification.

#### 3.2.2. H_2_-TPR

The decomposition of NO on Cu-ZSM-5 was considered as a Redox mechanism. NO can be decomposed continuously only when the redox cycle occurred between active copper species with different valence states. It was particularly important to study the change of copper species in catalysts [16]. There were different kinds of copper species in the Cu-ZSM-5 during exchanging process, and the migration of copper species occurred in the later heated process. Various types of copper species could coexist inside the Cu-ZSM-5; however, not all copper species had catalytic activity for NO decomposition. It was necessary to distinguish and quantify these copper species in the Cu-ZSM-5 [28].

**Figure 6:**
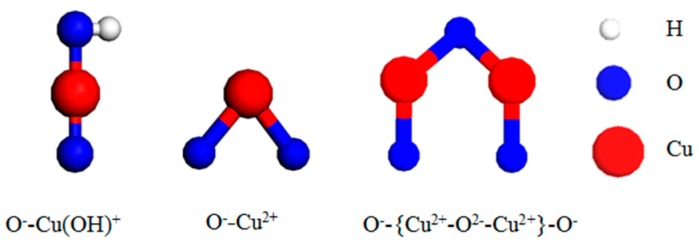
Copper Species in Cu-ZSM-5 catalyst.

The copper species in the Cu-ZSM-5 prepared in this experiment are shown in Figure 6. During the ion exchange, Cu^2+^ in the solution was hydrolyzed to form Cu(OH)^+^ [29]. Therefore the copper that entered ZSM-5 by exchanging NH_4_^+^ was mainly Cu^2+^ and Cu(OH)^+^ [30]. Cu(OH)^+^ falling at the exchange site being dehydrated to form isolated Cu^2+^ and {Cu^2+^-O^2−^-Cu^2+^}^2+^ dimer respectively with the OH^−^ group on the zeolite framework or another adjacent Cu(OH)^+^ during the subsequent heat treatment. Each of them then interacted with the ZSM-5 framework to balance the two positive charges [31,32], and the {Cu^2+^-O^2−^-Cu^2+^}^2+^ dimer was the active center of the NO catalytic decomposition reaction. XRD characterization of fresh Cu-ZSM-5 and Cu-Ce-ZSM-5 did not reveal the diffraction peak of CuO, indicating that there was no Cu^2+^ remaining on the surface through the multiple washing processes during the preparation of the catalyst. The subsequent heat treatment process also had no reducing atmosphere so that there were no Cu^+^ and Cu_2_O in the catalyst. The preparation process was always weakly acidic, so Cu(OH)_2_ precipitation would not form (when the pH was greater than 6, it was possible to form) [19].

**Figure 7:**
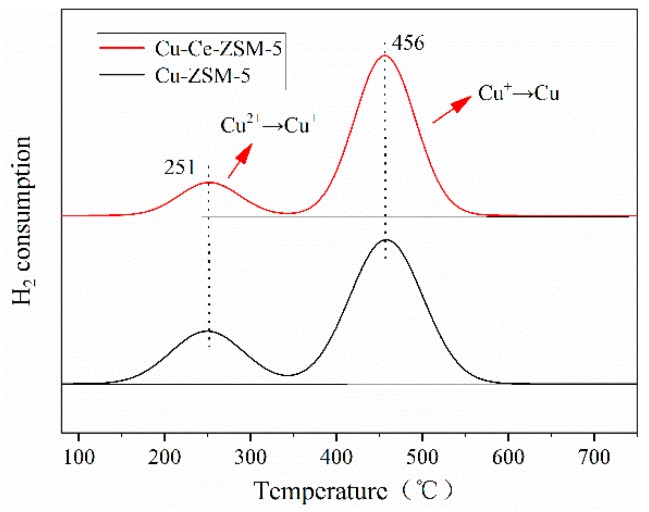
Characterization results of H_2_-TPR of fresh catalysts.

Figure 7 is the H_2_-TPR characterization result of Cu-ZSM-5 and Cu-Ce-ZSM-5. Two hydrogen- consumption peaks near 250 and 455 °C were detected, respectively. The reduction of standard CuO is a one-step reduction mechanism [33,34], as shown in Equation (2). At the same time, Cu^2+^ had been washed to prevent the formation of CuO, so these two hydrogen-consumption peaks have nothing to do with CuO. In fact, as shown in Equations (3) and (4), these two hydrogen-consumption peaks correspond to the two-step hydrogen consumption reduction of isolated Cu^2+^ and {Cu^2+^-O^2−^-Cu^2+^}^2+^ that formed from Cu(OH)^+^ in heat treatment process [35,36,37].

(2) $${\mathrm{CuO}+H}_{2}\to {\mathrm{Cu}}^{0}{+H}_{2}O$$

(3) Cu2++12H2→Cu++H+

(4) Cu++12H2→Cu0+H+

The lower temperature hydrogen-consuming peak was attributed to the reduction of bivalent copper to monovalent copper, while the higher temperature hydrogen-consuming peak was attributed to the reduction of monovalent copper to Cu^0^. Monovalent copper ions would not be introduced in the preparation of the catalyst, so the monovalent copper ions corresponding to the second hydrogen-consuming peak all came from the reduction of bivalent copper. Theoretically, the area of the two hydrogen-consuming peaks shall be basically the same, however, it was found that the area of the second peak was larger than that of the first peak due to the reason that the bivalent state {Cu^2+^-O^2−^-Cu^2+^}^2+^ was thermally unstable [38]. Pretreatment of samples at 500 °C for 4 h in Ar atmosphere before hydrogen temperature programmed reduction will make {Cu^2+^-O^2−^-Cu^2+^}^2+^ desorption O_2_, leading to high temperature self-reduction of this species and conversion of a monovalent {Cu^+^-□-Cu^+^}^2+^,as shown in Equation (5). According to this feature, {Cu^2+^-O^2−^-Cu^2+^}^2+^ had reduced to a monovalent {Cu^+^-□-Cu^+^}^2+^ without consuming hydrogen in the heating pretreatment stage before the temperature programmed reduction with hydrogen. Therefore, the first peak of hydrogen consumption should only correspond to the isolated Cu^2+^, while the second peak shall correspond to the monovalent copper formed by the hydrogen-consuming reduction of isolated Cu^2+^ and the self-reduction of {Cu^2+^-O^2−^-Cu^2+^}^2+^. Therefore, the area difference between the two hydrogen-consuming peaks could quantify the content of the active copper species {Cu^2+^-O^2−^-Cu^2+^}^2+^.

(5) $$2{\left\{{\mathrm{Cu}}^{2+}{\text{-}O}^{2\text{-}}{\text{-}\mathrm{Cu}}^{2+}\right\}}^{2+}\to 2{\left\{{\mathrm{Cu}}^{+}\text{-}□{\text{-}\mathrm{Cu}}^{+}\right\}}^{2+}{+O}_{2}$$

The difference of the two hydrogen-consuming peaks of Cu-Ce-ZSM-5 was larger than that of Cu-ZSM-5, indicating that Ce doping could improve the relative content of {Cu^2+^-O^2−^-Cu^2+^}^2+^ in the catalyst. This was mainly because Ce promoted the dispersion and prevented aggregation of Cu(OH)^+^ in copper nitrate solution [39]; it ensured that more Cu(OH)^+^ had the opportunity to enter the channel of ZSM-5 and exchange with NH_4_^+^. Cu(OH)^+^ was the precursor of the active center {Cu^2+^-O^2−^-Cu^2+^}^2+^. The increase of {Cu^2+^-O^2−^-Cu^2+^}^2+^ corresponded to the increase of catalyst activity in NO catalytic decomposition evaluation test. The doped Ce also competed with Cu^2+^ for exchange sites, resulting in a decrease in exchange capacity of inactive Cu^2+^.

**Figure 8:**
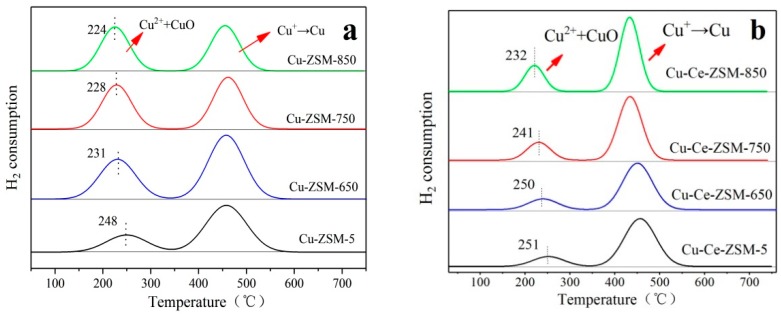
Characterization results of H_2_-TPR of different catalysts. (**a**) Cu-ZSM-5 and Cu-ZSM-X. (**b**) Cu-Ce-ZSM-5 and Cu-Ce-ZSM-X.

Figure 8 shows H_2_-TPR results of fresh Cu-ZSM-5 and Cu-Ce-ZSM-5 and aged Cu-ZSM-X and Cu-Ce-ZSM-X. In the Cu-ZSM-X sample, it is considered that the first hydrogen consuming peak shifts toward the low temperature direction, the variation range is 17–24 °C. Compared with the Cu-ZSM-X, it can be found that the decrease of temperature corresponding to the first peak of the Cu-Ce-ZSM-X is lower than that of the Cu-ZSM-X. The decrease of temperature for the first hydrogen-consuming peak indicates that this peak no longer fully represents the hydrogen consumption of isolated Cu^2+^. The reduction temperature of CuO has been proved to be lower than that of isolated Cu^2+^. Combined with the results of XRD that little CuO was detected in the sample with the increase of hydrothermal aging temperature, the temperature for the first hydrogen-consuming peak shifted to a lower temperature indicating that the peak also included hydrogen consumption caused by a small amount of CuO. The results of H_2_-TPR characterization illuminated that the isolated Cu^2+^ and the active center {Cu^2+^-O^2−^-Cu^2+^}^2+^ in the zeolite would migrate and form inactive CuO.

Although the reduction temperature of CuO is lower than that of isolated Cu^2+^, the difference between the two temperatures is small. CuO and isolated Cu^2+^ superimposed on the hydrogen consumption to form the first peak. It is difficult to separate the two independent hydrogen-consuming peaks to investigate the relative content of CuO in Cu-ZSM-X and Cu-Ce-ZSM-X. However, since CuO is a one-step reduction mechanism, the second hydrogen-consuming peak still can be attributed to monovalent copper only from the reduction of isolated Cu^2+^ ions and {Cu^2+^-O^2−^-Cu^2+^}^2+^. The ratio of the area for this second peak to the total hydrogen-consuming peak area can represent the total content of isolated Cu^2+^ and {Cu^2+^-O^2−^-Cu^2+^}^2+^, and the magnitude of the decrease in this ratio also represents the amplitude of the increase in CuO content.

Table 3 showed the relative proportion of the two hydrogen-consuming peaks in Cu-ZSM-5 and Cu-Ce-ZSM-5 and hydrothermal aged Cu-ZSM-X and Cu-Ce-ZSM-X. The area of the second hydrogen-consuming peak of the Cu-ZSM-5 accounted for 73.1% of the total peak area. With the process of hydrothermal aging treatment and the increase of treatment temperature, the ratio of the area of the second hydrogen-consuming peak decreased 15.9%, 26.0%, and 30.6%, respectively. It indicates that the higher the hydrothermal aging temperature, the more severe the isolated Cu^2+^ ions and {Cu^2+^-O^2−^-Cu^2+^}^2+^ migration, and the more CuO is formed. For the modified Cu-Ce-ZSM-5, the area of the second hydrogen-consumin peak accounted for 83.7% of the total peak area. With the increase of hydrothermal aging temperature, the area proportion of the second hydrogen-consumin peak also decreased, but the decrease range was significantly less than that of the unmodified Cu-ZSM-X. The Cu-Ce-ZSM-850 was only reduced by 11.8% compared to the Cu-Ce-ZSM-5, indicating that the Ce modification can effectively inhibit the process that isolated Cu^2+^ and {Cu^2+^-O^2−^-Cu^2+^}^2+^ migrate to CuO.

At present, it is believed that there are two cation exchange sites in the ZSM-5, which are bridging hydroxyl groups and aluminum hydroxyl groups. The cation coordination with the bridging hydroxyl group has a poor stability [40], the doped Ce can occupy the bridging hydroxyl group preferentially, and the copper ion occupies more stable aluminum hydroxyl group, so it is not easy to migrate in the process of hydrothermal aging treatment and reflect higher hydrothermal aging resistance.

#### 3.2.3. XPS

**Figure 9:**
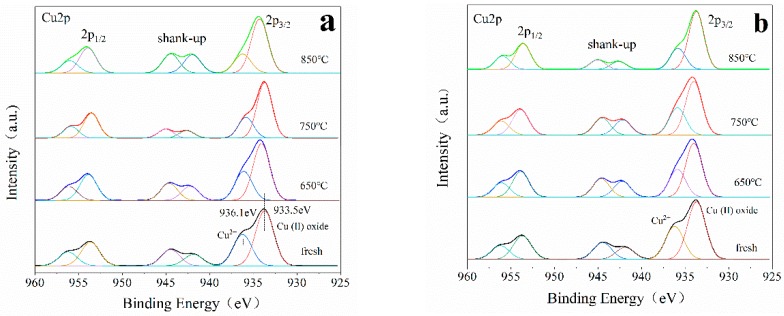
XPS spectra of Cu2p of different catalysts. (**a**) Cu-ZSM-5 and Cu-ZSM-X. (**b**) Cu-Ce-ZSM-5 and Cu-Ce-ZSM-X.

The chemical state and surface composition of the elements in the catalyst were characterized by XPS. Figure 9 shows the XPS results of Cu2p of unmodified Cu-ZSM-5 and Cu-ZSM-X and doped Ce modified Cu-Ce-ZSM-5 and Cu-Ce-ZSM-X. All samples have two main absorption peaks: the first peak at 931–938 eV belongs to the Cu2p^3/2^, the second peak at 951–958 eV belongs to the Cu2p^1/2^. A strong satellite peak near 941–946.5 eV confirmed the existence of bivalent copper. Monovalent copper only has a weak satellite peak at 945 eV, and the peak width of monovalent copper and Cu^0^ in the Cu2p^3/2^ is also narrow, indicating that there is no monovalent copper and Cu^0^ in these catalysts [41]. After peak-fitting, the Cu2p^3/2^ absorption peak can be well fitted to two absorption peaks of 933.5 and 936.1 eV. The former peak corresponding to the oxidation state of bivalent copper, that is the active center {Cu^2+^-O^2−^-Cu^2+^}^2+^ and CuO formed by migration after hydrothermal aging treatment [41,42]. The latter being the signal peak of the isolated Cu^2+^ ion corresponds to the oxygen atom in the ZSM-5 [43,44]. The absorption peak at 933.5 eV in the fresh Cu-ZSM-5 and Cu-Ce-ZSM-5 should not contain CuO, but only contain {Cu^2+^-O^2−^-Cu^2+^}^2+^. The absorption peak area at 933.5 eV is larger than 936.1 eV, indicating that the content of {Cu^2+^-O^2−^-Cu^2+^}^2+^ in fresh samples is higher than isolated Cu^2+^. With the increase of hydrothermal aging temperature, the signal peak of isolated Cu^2+^ decreases gradually, indicating that part of isolated Cu^2+^ is converted into CuO. The reduced amplitude of the signal peak of isolated Cu^2+^ in the Ce-modified catalyst decreases, which proves that the doping of Ce can slow down the migration process of forming CuO.

#### 3.2.4. NO-TPD

**Figure 10:**
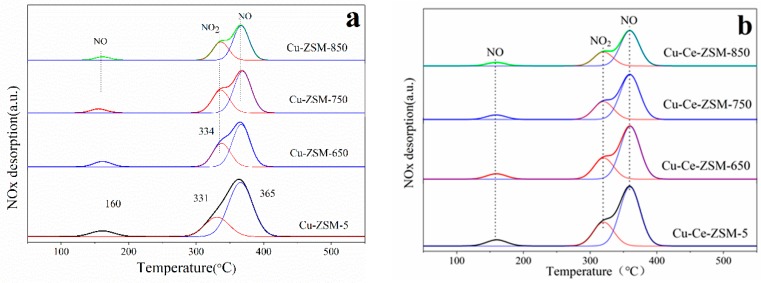
Characterization results of NO-TPD of different catalysts. (**a**) Cu-ZSM-5 and Cu-ZSM-X. (**b**) Cu-Ce-ZSM-5 and Cu-Ce-ZSM-X.

The characterization of NO-TPD can help to understand the adsorption capacity of reactive species on the surface of catalyst. Figure 10 shows the NO-TPD results of fresh Cu-ZSM-5 and Cu-Ce-ZSM-5 and other samples aged by different hydrothermal temperature. After the peak-fitting, three NO_x_ desorption peaks appeared in all samples. The NO adsorbed on the surface of the catalyst in the form of weak physical adsorption was completely desorbed in the purge stage before temperature programmed desorption, so these three desorption peaks are independent of that process. The desorption peak at 160 °C was attributed to NO that desorbed from the isolated Cu^2+^ [36,37], and the desorption peak of NO_2_ near 334 °C corresponded to the reaction between NO and the active center {Cu^2+^-O^2−^-Cu^2+^}^2+^ [36], as shown in Equation (6). The desorption of NO near 365 °C was attributed to the thermal decomposition of nitrate or nitrite generated by NO adsorption on {Cu^2+^-O^2−^-Cu^2+^}^2+^ [16,45].

(6) $$\mathrm{NO}+{\left\{{\mathrm{Cu}}^{2+}{\text{-}O}^{2\text{-}}{\text{-}\mathrm{Cu}}^{2+}\right\}}^{2+}\to {\mathrm{NO}}_{2}+{\left\{{\mathrm{Cu}}^{+}\text{-}□{\text{-}\mathrm{Cu}}^{+}\right\}}^{2+}$$

**Figure 11:**
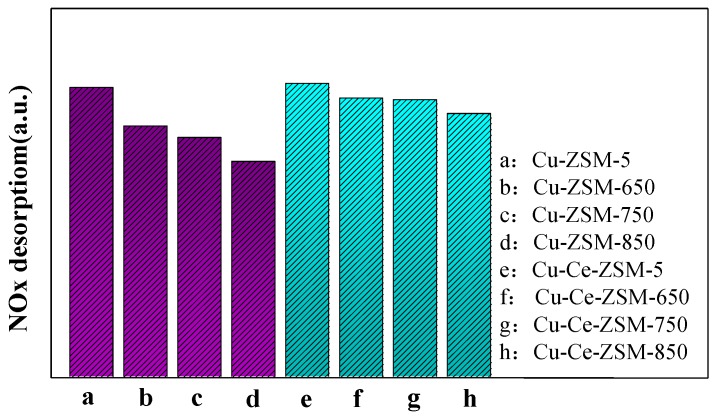
Total desorption amount of the last two NO_x_ desorption peaks related to {Cu^2+^-O^2−^-Cu^2+^}^2+^ in fresh catalyst and hydrothermal aging catalyst. (**a**) Cu-ZSM-5 and Cu-ZSM-X. (**b**) Cu-Ce-ZSM-5 and Cu-Ce-ZSM-X.

The NO_x_ desorption of the modified Cu-Ce-ZSM-5 and Cu-Ce-ZSM-X was similar to that of Cu-ZSM-5 and Cu-ZSM-X. It can be found that the last two desorption processes of NO_x_ are related to the activity center {Cu^2+^-O^2−^-Cu^2+^}^2+^. The integral area summation of the last two NO_x_ desorption peaks of all samples was shown in Figure 11. The total integral area of the second and third NO_x_ desorption peaks of modified and unmodified samples all showed a gradual decrease trend. It indicates that {Cu^2+^-O^2−^-Cu^2+^}^2+^ decreases in the samples after hydrothermal aging treatment, and the decrease amplitude enhances with the increase of hydrothermal aging temperature. According to the characterization results of H_2_-TPR, it can be inferred that this is due to the migration of {Cu^2+^-O^2−^-Cu^2+^}^2+^ to form CuO. Meanwhile, the NO_x_ desorption of Cu-Ce-ZSM-X was also lower than that of Cu-Ce-ZSM-5, but with the increase of hydrothermal aging temperature, the decrease range is smaller than that of the unmodified Cu-ZSM-X. This also confirmed the conclusion in the H_2_-TPR characterization that the Ce modification can alleviate the decrease of the active copper species {Cu^2+^-O^2−^-Cu^2+^}^2+^.

**Figure 12:**
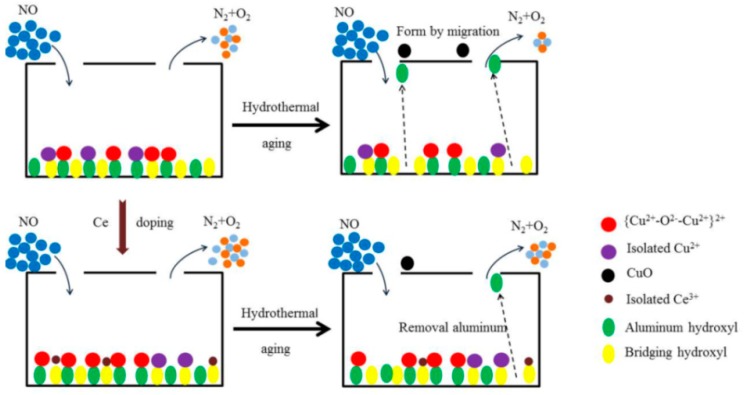
Deactivation and modification mechanism of Cu-ZSM-5 catalyst.

According to experiment and characterization results, the main reasons for the deactivation of Cu-ZSM-5 caused by hydrothermal aging include physical and chemical aspects. It leads to deterioration of the structure and morphology of the catalyst in physical aspect, making the active center migrate to inert species in chemical aspect. The modification of doped Ce mainly alleviates the adverse chemical transformation. The deactivation mechanism and modification mechanism of hydrothermal aging of catalyst are shown in Figure 12.

## 4. Conclusions

(1) The catalytic activity of Cu-ZSM-5 series catalysts was inhibited to some extent in the reaction atmosphere containing water vapor. It was also decreased after 10 h of hydrothermal aging treatment at 650–850 °C, and the deactivation of the sample treated at 850 °C was the most serious.

(2) Hydrothermal aging treatment reduced the crystallinity of Cu-ZSM-5 series catalysts. The diffraction peak intensity of the sample aged at 850 °C decreased obviously. The CuO was formed in some samples after high-temperature hydrothermal aging.

(3) The pore structure characterization showed that high-temperature hydrothermal aging treatment would lead to dealumination of the ZSM-5. The content of Cu(OH)^+^ exchange site decreases. The exfoliated non-framework aluminum covers and blocks the micropore, reducing the specific surface area and pore volume of the micropore. At the same time, the structure of zeolite framework was destroyed and mesopore appeared. The specific surface area and pore volume of micropore affect the activity of catalysts.

(4) Cu-ZSM-5 series catalysts coexist with isolated Cu^2+^ and active center {Cu^2+^-O^2−^-Cu^2+^}^2+^; hydrothermal aging treatment will cause the migration of {Cu^2+^-O^2−^-Cu^2+^}^2+^ in the catalyst channel and the formation of inactive CuO. The increasing temperature of hydrothermal aging will aggravate the migration process.

(5) Doping Ce can improve the ability of Cu-ZSM-5 catalyst to against water vapor and hydrothermal aging effect. The physical properties of the modified catalyst and the unmodified catalyst are very similar, doping Ce will not improve the framework strength and pore structure. The chemical properties of the modified catalyst are significantly different from those of the unmodified catalyst, so the effect of modification doped Ce is mainly reflected in chemistry aspect. On the one hand, Ce promotes the dispersion of Cu(OH)^+^ during the exchange process and competes with Cu^2+^ for exchange sites. On the other hand, it makes the Cu(OH)^+^ exchange at a more stable and active aluminum hydroxyl position, thereby inhibiting the migration of active copper species caused by hydrothermal aging effect.

## Figures and Tables

**Table 1:** Copper and cerium content of Cu-ZSM-5 and Cu-Ce-ZSM-5.

Sample	Cu ^a^ wt. %	Al ^a^ wt. %	Cu Exchange Degree ^b/^%	Ce ^a^ wt. %
Cu-ZSM-5	1.75	2.86	51.6	0
Cu-Ce-ZSM-5	1.69	2.90	49.2	0.027

^a^ content detected by ICP-OES. ^b^ Twice the molar ratio of Cu to Al atoms. The element Al can be accurately measured and has the same number of moles as the monovalent cation.

**Table 2:** BET results of Cu-ZSM-5 and Cu-Ce-ZSM-5 catalysts treated with hydrothermal aging at different temperatures.

Sample	Surface Area/(m^2^/g)				Volume/(cm^3^/g)			Pore Size/nm
	BET	Micropore	Percent	Mesopore	Total	Micropore	Percent	
Cu-ZSM-5	316.6	241.2	76.2%	75.4	0.170	0.121	71.5%	2.145
Cu-ZSM-650	324.5	100.5	31.0%	224.0	0.177	0.055	31.4%	2.181
Cu-ZSM-750	295.9	97.7	33.0%	198.2	0.169	0.050	29.7%	2.287
Cu-ZSM-850	180.1	82.6	45.9%	97.5	0.120	0.041	35.0%	2.664
Cu-Ce-ZSM-5	309.5	238.0	76.9%	71.5	0.164	0.120	72.9%	2.124
Cu-Ce-ZSM-650	325.4	106.2	32.6%	219.2	0.175	0.058	33.2%	2.159
Cu-Ce-ZSM-750	273.7	99.8	36.5%	173.9	0.163	0.051	31.1%	2.384
Cu-Ce-ZSM-850	176.6	83.9	47.5%	92.7	0.126	0.042	33.3%	2.629

**Table 3:** Relative proportion of the first and second hydrogen-consuming peak area of unmodified and modified catalysts (%).

Sample	The first Reduction Peak Area Ratio	The Second Reduction Peak Area Ratio
Cu-ZSM-5	26.9	73.1
Cu-ZSM-650	38.5	61.5
Cu-ZSM-750	45.9	54.1
Cu-ZSM-850	49.3	50.7
Cu-Ce-ZSM-5	16.3	83.7
Cu-Ce-ZSM-650	18.9	81.1
Cu-Ce-ZSM-750	21.9	78.1
Cu-Ce-ZSM-850	26.2	73.8

## Supplementary Materials

The following are available online: https://www.mdpi.com/1996-1944/13/4/888/s1, Figure S1: SEM images of fresh samples and aged samples treated with different hydrothermal aging temperatures. A represents the unmodified sample and B represents the Ce modified sample. The number 1 represents fresh samples, and the numbers 2, 3, and 4 represent samples subjected to hydrothermal aging at 650, 750, and 850 °C, respectively, Figure S2: Aspirating desorption curves of different catalysts. (a) Cu-ZSM-5 and Cu-ZSM-X. (b) Cu-Ce-ZSM-5 and Cu-Ce-ZSM-X, Figure S3: Results of pore size distributions of fresh catalysts and hydrothermally aged catalysts obtained from low temperature nitrogen physical adsorption experiments (a) Cu-ZSM-5 and Cu-ZSM-X. (b) Cu-Ce-ZSM-5 and Cu-Ce-ZSM-X.
