# Supplementary material for: Effect of Hydrothermal Aging Treatment on Decomposition of NO by Cu-ZSM-5 and Modified Mechanism of Doping Ce against This Influence
Source: Materials (Basel). 2020 Feb 17;13(4):888. doi: 10.3390/ma13040888 (PMC7079666; doi:10.3390/ma13040888)
Supplement: Supplementary file 1 [file materials-13-00888-s001.pdf]

Supplementary Materials: Effect of Hydrothermal Aging Treatment on Decomposition of NO by Cu-ZSM-5 and Modified Mechanism of Doping Ce against This Influence

Xiao Yang, Xiaofei Wang, Xiaolei Qiao, Yan Jin and Baoguo Fan *

College of Electrical and Power Engineering, Taiyuan University of Technology, Yingze West Street 79, Taiyuan 030024, China; yangxiao0165@link.tyut.edu.cn (X.Y.); wangxiaofei0167@link.tyut.edu.cn (X.W.); qiaoxiaolei@tyut.edu.cn (X.Q.); jinyan@tyut.edu.cn (Y.J.)

* Correspondence: fanbaoguo@tsinghua.org.cn.

1. SEM Images

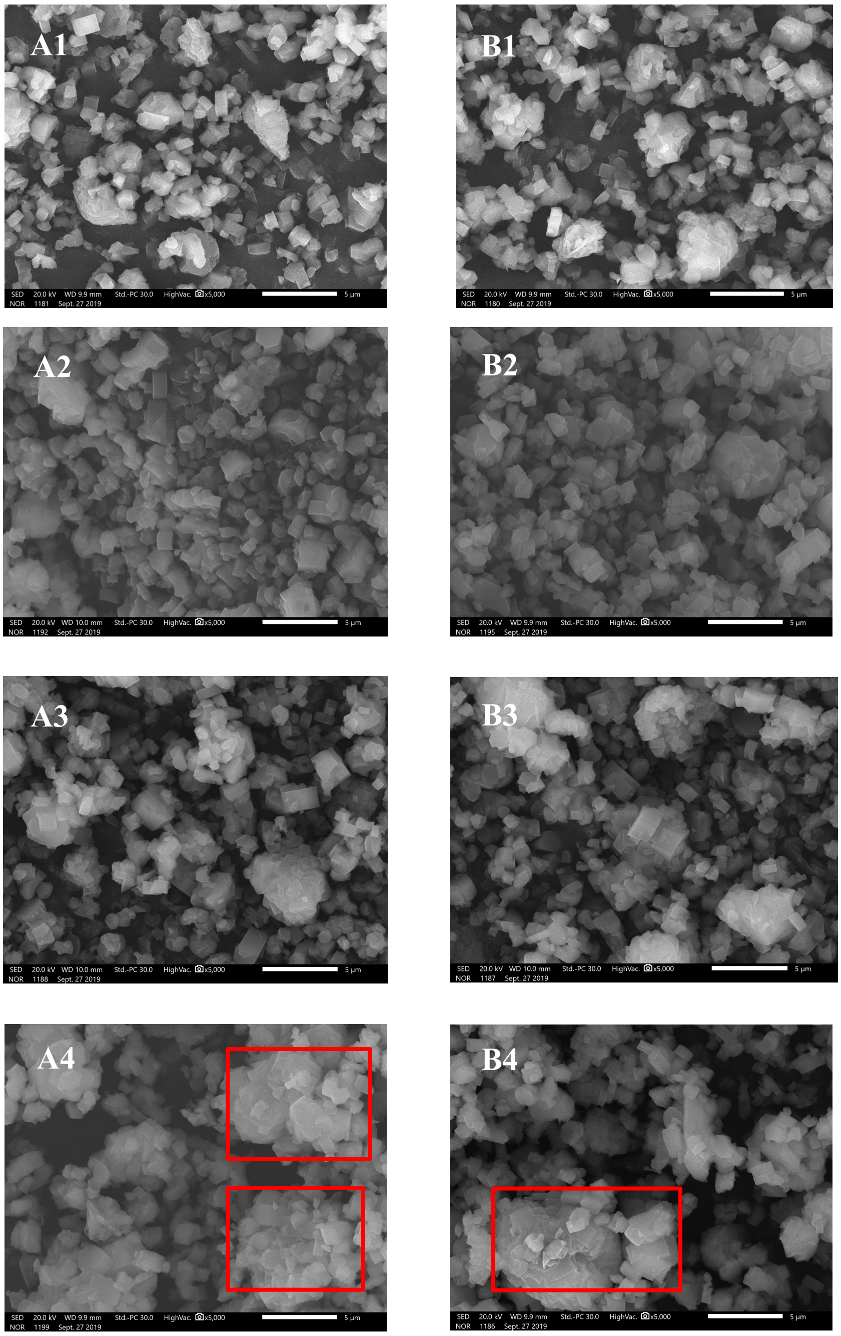

Figure S1. SEM images of fresh samples and aged samples treated with different hydrothermal aging temperatures. A represents the unmodified sample and B represents the Ce modified sample. The number 1 represents fresh samples, and the numbers 2, 3, and 4 represent samples subjected to hydrothermal aging at 650, 750, and 850 °C, respectively.

The magnification was 5k times. It could be seen from the image that the unmodified and modified catalysts had very similar trends in surface morphology as the hydrothermal aging temperature increased. In the fresh samples, the crystallites of zeolite were relatively dispersed and had a high degree of crystallinity, and the profile was clear. As the temperature of the hydrothermal aging treatment increased, samples were treated at 650 and 750 °C partially agglomerated, but the zeolite profile was still distinguishable. However, when the hydrothermal aging temperature was 850 °C, it could be clearly seen that the catalyst particles appeared a very serious agglomeration phenomenon, as marked by the rectangle. The zeolite crystallites were also partially destroyed, the profile was no longer clear, and the crystallinity decreased significantly. It is consistent with the XRD characterization results above. This indicated that the hydrothermal aging treatment leads to agglomerate tend of the sample, but significant agglomeration and crystallization deterioration occured only when the temperature reached 850 °C, and the agglomeration phenomenon at 650 and 750 °C was not serious. It was consistent with the activity evaluation result of the decomposition of NO by the catalyst.

2. The Adsorption Desorption Curves

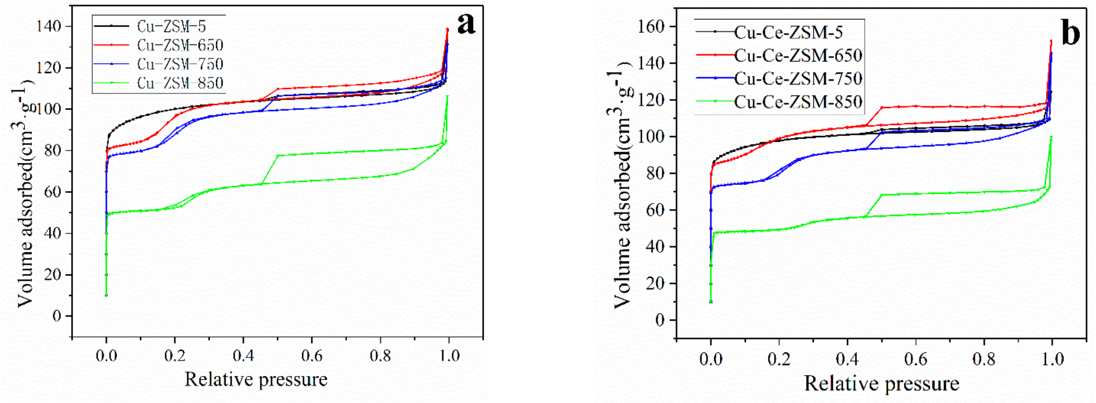

Figure S2. Adsorption desorption curves of different catalysts. (**a**) Cu-ZSM-5 and Cu-ZSM-X. (**b**) Cu-Ce-ZSM-5 and Cu-Ce-ZSM-X.

The relative pressure $p/p_0 = 0$ nearby showed the micropore filled adsorption that having a diameter less than 2 nm. The unmodified and modified samples showed similar trends in the micropore adsorption, and the adsorption amount both decreased with the increase of hydrothermal aging temperature. The decrease of samples treated at 650 and 750 °C were approximate, and the decrease at 850 °C was most pronounced. Cu-ZSM-750 and Cu-ZSM-850 and Cu-Ce-ZSM-750 samples began to appear hysteresis loop around $p/p_0 = 0.17$, which showed the transition from submicropore to mesopore. All of the hydrothermal aging treated samples showed obvious hysteresis loop at $p/p_0 = 0.45$, and it was a typical H1 type hysteresis loop. The appearance of hysteresis loop indicated that a large number of mesopores formed in these samples [28].

3. The Pore Size Distributions

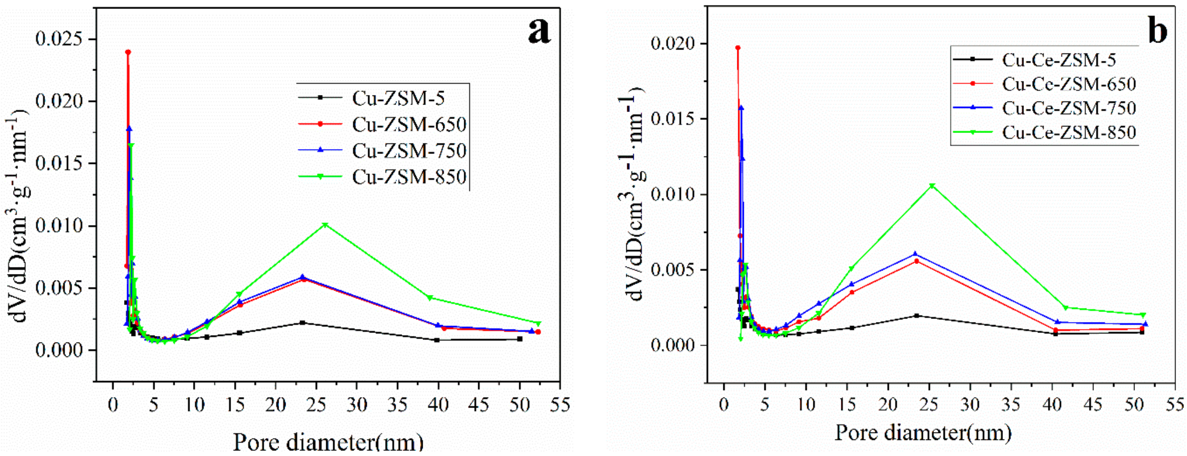

Figure S3. Results of pore size distributions of fresh catalysts and hydrothermally aged catalysts obtained from low temperature nitrogen physical adsorption experiments (**a**) Cu-ZSM-5 and Cu-ZSM-X. (**b**) Cu-Ce-ZSM-5 and Cu-Ce-ZSM-X.

© 2020 by the authors. Submitted for possible open access publication under the terms and conditions of the Creative Commons Attribution (CC BY) license (http://creativecommons.org/licenses/by/4.0/).
